# Transglutaminase activity in the hematopoietic tissue of a crustacean, *Pacifastacus leniusculus*, importance in hemocyte homeostasis

**DOI:** 10.1186/1471-2172-9-58

**Published:** 2008-10-07

**Authors:** Xionghui Lin, Kenneth Söderhäll, Irene Söderhäll

**Affiliations:** 1 Department of Comparative Physiology, Uppsala University, Norbyvägen 18A, SE-752 36 Uppsala, Sweden

## Abstract

**Background:**

Transglutaminases (TGases) form a group of enzymes that have many different substrates and among the most well known are fibrin for Factor XIIIa and the clotting protein in crustaceans. We also found that TGase is an abundant protein in the hematopoietic tissue (Hpt) cells of crayfish and hence we have studied the possible function of this enzyme in hematopoiesis.

**Results:**

TGase is one of the most abundant proteins in the Hpt and its mRNA expression as well as enzyme activity is very high in the Hpt cells, lesser in the semi-granular hemocytes and very low in the granular cells. In cultured hematopoietic tissues, high activity was present in cells in the centre of the tissue, whereas cells migrating out of the tissue had very low TGase activity. RNAi experiments using dsRNA for TGase completely knocked down the transcript and as a result the cell morphology was changed and the cells started to spread intensely. If astakine, a cytokine directly involved in hematopoiesis, was added the cells started to spread and adopt a morphology similar to that observed after RNAi of TGase. Astakine had no effect on TGase expression, but after a prolonged incubation for one week with this invertebrate cytokine, TGase activity inside and outside the cells was completely lost. Thus it seems as if astakine addition to the Hpt cells and RNAi of TGase in the cell culture will lead to the same results, i.e. loss of TGase activity in the cells and they start to differentiate and spread.

**Conclusion:**

The results of this study suggest that TGase is important for keeping the Hpt cells in an undifferentiated stage inside the hematopoietic tissue and if expression of TGase mRNA is blocked the cells start to differentiate and spread.

This shows a new function for transglutaminase in preventing hematopoietic stem cells from starting to differentiate and migrate into the hemolymph, whereas their proliferation is unaffected. Astakine is also important for the hematopoiesis, since it induces hemocyte synthesis in the Hpt but now we also show that it in some unknown way participates in the differentiation of the Hpt cells.

## Background

Transglutaminases (TGases) form a family of Ca^2+^-dependent enzymes catalyzing post-translational remodelling of proteins such as, cross-linking in homeostasis, keratinocyte cornified envelope formation and semen coagulation [1-3]. These enzymes catalyze a R-glutaminyl-peptide:amine-γ-glutamyl-transferase reaction leading to cross-linking within or between polypeptide chains, or covalent incorporation of polyamines into protein substrates. In crustaceans, TGases are mainly known to be involved in the plasma clotting reaction [4-9] as mammalian plasma factor XIIIa. In the horseshoe crab TGase and its clotting substrate proxin are both located in the amoebocytes and after release into the plasma TGase promotes cross-linking of the cell surface proxin to coagulin, whereas in crayfish TGase located in hemocytes are released upon activation and catalyzes the cross-linking of a high density lipoprotein in plasma named the clotting protein [4,7,8,10]. A similar clotting reaction was also detected in different shrimp species [11]. The horseshoe crab TGase was recently also found to participate in the host defence in the cuticle by cross-linking Caraxin-1, a component of cuticle, into a stable mesh, which promotes wound healing and sclerotization at injured sites of the cuticle [12].

All crustacean TGases known so far show highest similarity with mammalian plasma factor XIIIa, and we have also confirmed this similarity in the promoter region (GenBank accession EU195879). Factor XIIIa participates in the final step of plasma coagulation in mammals by catalyzing the formation of cross-links between fibrin molecules to yield a stabilized clot [13]. However, more recent research has revealed new functions for Factor XIIIa in stimulating angiogenesis and in tissue repair [14]. The plasma form of Factor XIIIa is produced by cells of bone marrow origin such as monocytes, macrophages, megakaryocytes and platelets, and the intracellular form was also found in the bone marrow precursors of monocytes and megakaryocytes, but its function is not yet fully understood [15]. In osteoblasts and osteocytes, surface Factor XIIIa was found to have a more prominent role in matrix formation than TG2, which contributes to later mineralization of osteoblasts cultures [16]. In the tiger shrimp *P. monodon *high expression of shrimp transglutaminase (shrimp TG) was detected in young hemocytes in the hematopoietic tissue. In situ hybridization, especially dividing cells and cells with more condensed cytoplasm always yielded stronger TG signals [17]. In the present study, we show that crayfish TGase, a homologue of the mammalian Plasma Factor XIIIa, is one of the most abundant proteins of the hematopoietic tissue, its activity was found both inside and on the surface of the hematopoietic tissue (Hpt) cells. The Hpt cells of freshwater crayfish have been used as a complement to in vivo studies to study the in vitro immune response these cells mount towards infection with a virus, the white spot syndrome virus, or a bacterium, *Aeromonas hydrophila *[18,19]. We also found that down regulation by RNAi in the Hpt cell culture resulted in a morphology change and loss of TGase activity on the cell surface, indicating that TGase plays a role in the hematopoietic tissue in this crustacean.

## Methods

### Experimental animals and preparation of hematopoietic tissue cells

Freshwater crayfish, *Pacifastacus leniusculus*, were purchased from Torsäng, lake Vättern, Sweden, and kept in aquaria in aerated tap water at 10°C. Only intermoult animals were used.

The crayfish hematopoietic tissue cells were isolated according to Söderhäll et al. [20] with minor modifications. The hematopoietic tissue was dissected from the dorsal side of the stomach, and was incubated in 700 μl of 0.1% collagenase (Type I), 0.1% collagenase (Type IV) in crayfish phosphate buffered saline (CPBS) (10 mM Na_2_HPO_4_, 10 mM KH_2_PO_4_, 0.15 M NaCl, 10 μM CaCl_2_, 10 μM MnCl_2_; pH 6.8) at room temperature for 40 min. After collagenase treatment the tissue was centrifuged at 2000 × g for 5 min to remove the collagenase solution. The pellet was washed twice with 1 ml CPBS and the undigested tissues was removed, and the isolated Hpt cells were resuspended in modified L-15 medium [21], and subsequently seeded in 96 well or 6 well plates at a density of 5 × 10^5 ^cells/ml. The Hpt cells were supplemented with or without a crude astakine preparation [21] after 1 hr of attachment at room temperature, and one third of the medium was changed every second day.

For tissue culture, the remaining undigested tissue after preparing the cell cultures was resuspended in 2 ml L-15 medium, and then cultured on coverslips in six well plates, and one half of the medium was changed every second day.

### Separation of semi-granular and granular hemocytes

Isolated populations of crayfish semi-granular and granular hemocytes were obtained as earlier described [22]. Briefly, 1–2 ml hemolymph was collected in 2 ml anticoagulant buffer (0.14 M NaCl, 0.1 M glucose, 30 mM trisodium citrate, 26 mM citric acid, 10 mM EDTA, pH 4.6) [22], and subsequently centrifuged at 1,700 g for 20 min through preformed continuous gradients of 70% Percoll (GE, Uppsala, Sweden) in 0.15 M NaCl. The resulting cell bands were collected separately to prepare total RNA.

### Preparation of dsRNA

Oligonucleotide primers were designed to amplify a 503-bp region of the *P. leniusculus *TGase gene, which was used as a template to synthesize the dsRNA of TGase, and T7 promoter sequences (italics) was appended at the 5' end of the primers: 895+, 5'-*taatacgactcactataggg*tcttcgggcagtttg-3'; 1398-, 5'-*taatacgactcactataggg*ccgccatag ccatcagg-3'. The template was generated by PCR using primers specific for portions of the GFP gene from pd2EGFP-1 vector (Clontech, Palo Alto, CA, USA), which was used to prepare the control dsRNA [23], and the primers were: 63+5'*taatacgactcactataggg*cgacgtaaacggccacaagt3', 719-5'*taatacgactcactataggg *ttcttgtacagct cgtccatgc 3'. To generate dsRNA, 1 μg PCR product was purified by gel extraction (Qiagen, Hilden, Germany) and used as template for *in vitro *transcription according to the manual of the MegaScript kit (Ambion, Austin, TX, USA), and the dsRNAs were purified with Trizol^® ^LS Reagent (Invitrogen, Carlsbad, CA, USA).

### RNAi *in vitro*

Transfection of dsRNA was made according to Liu and Söderhäll [23] with minor modifications. Briefly, 4 μl dsRNA (250 ng/μl) was mixed with 3 μl calf histone H2A (histone from calf thymus, Type II-A, 1 mg/ml dissolved in modified L-15 medium) (Sigma, Steinheim, Germany) for each well of the Hpt cell cultures and incubated for 5–10 min at room temperature followed by mixing with 20 μl modified L-15 medium, and subsequently added to the newly prepared Hpt cell cultures. One-third of the total volume of medium was changed every second day during incubation of the Hpt cell cultures.

### Total RNA preparation and RT-PCR

Total RNA was extracted from the cultured Hpt cells or hemocytes by using Gene Elute Total Mammalian RNA extraction kit (Sigma-Aldrich), followed by RNase free DNase I (Ambion, Austin, TX, USA) treatment. Equal amounts of total RNA was used for cDNA synthesis with ThermoScript™ (Invitrogen, Carlsbad, CA, USA) according to the manufacture's instructions and analyzed for expression of crayfish TGase by RT-PCR using the following primers: 5'-tgggycttcgggcagtt-3'; 5'-cgaagggcacgtcgtac-3'. The PCR program used was as follows: 94°C, 3 min, followed by 30 cycles of 94°C for 30 s, 60°C for 30 s, 72°C for 40 s and the transcription of a 40S ribosomal protein was used as a control. All PCR products were analyzed on 1.5% agarose gel stained with ethidium bromide.

### Cloning of the promoter region of the TGase gene

Genomic DNA of crayfish was isolated from the hemocytes according to the manual instruction of GenElute TM Blood Genomic DNA Kit. The promoter region of the TGase gene was cloned by GenomeWalker TM Universal Kit (BD Biosciences, Clontech) according to the manufacturer's instructions. Briefly, separate aliquots of genomic DNA (2.5 μg) was digested completely with Dra I, EcoRV, PvuII and StuI, and each batch of digested genomic DNA was purified and ligated separately to the genomeWalker Adaptor to construct four genomic libraries. Two specific primers corresponding to 5' coding region of the TGase gene was designed as follows: TGSP1: 5'-agcctcaatctcgttctctaaagc-3'; TGSP2: 5'-gttctctatacgttcaaagacagc-3', then nested PCR was performed by using DNA from the four libraries as templates separately, primary PCRs were carried out with TGSP1 and AP1 (Clontech adaptor primer). Secondary nested PCRs were performed with GSP2 and AP2 (Clontech nested adaptor primer), and the PCR products was cloned to TA vector for sequencing.

### Preparation of whole cell lysates and TGase activity assay

Freshly prepared Hpt cells or separated hemocytes were homogenized in 500 ul RIPA buffer (50 mM Tris, 150 mM NaCl, 10 mM EDTA, 1% NP-40,0.1% SDS, pH 7.5), containing 1× protease inhibitor cocktail (Complete Mini, Roche Diagnostics, Mannheim, Germany). The different homogenates were then each centrifuged at 20,000 × *g *for 15 min at 4°C, and the resulting whole cell lysates were used in the TGase activity assay. The protein concentration of the whole cell lysate was determined by 2-D Quant kit (GE Healthcare). Each sample was assayed in triplicate.

The TGase activity of whole cell lysates was determined by a modified non-radioactive microtiter plate assay [24]. Briefly, the microtiter plates were coated with 200 μl of *N,N*'-dimethylcasein (Sigma, 10–20 mg/ml) at 4°C overnight, and the wells were blocked with non-fat dry milk (0.5% in 0.1 M Tris-HCl, pH 8.5) for 30 min. and washed two times with 350 μl of 0.1 M Tris-HCl, pH 8.5. Reagents were added to each well as follows: 5 mM CaCl_2_, 10 mM dithiothreitol, 0.5 mM 5' (biotinamido)-pentylamine (Pierce), 20 μg whole cell lysates, and 0.1 M Tris-HCl, pH 8.5, to obtain a total volume of 200 μl per well. The microtiter plate was incubated for 30 min at 37°C, and the liquid was discarded and then the reaction was stopped by washing twice with 350 μl of EDTA (200 mM), followed by washing twice with 350 μl of 0.1 M Tris-HCl, pH 8.5. The Streptavidin-Horseradish Peroxidase conjugates were diluted 1:200 with non-fat dry milk (0.5% in 0.1 M Tris-HCl, pH 8.5) prior to adding 250 μl of the solution per well for a l h incubation at room temperature. The plate was washed once with 350 μl of 0.001% Triton X-100 followed by four washes with 350 μl 0.1 M Tris-HCl, pH 8.5. Then 200 μl of 0.1 M Tris-HCl, pH 8.5, and 200 μl of substrate solution (TMB, Sigma T0440) were added to each well. After incubation for 10–20 min at room temperature, the reactions were stopped by the addition of 50 μl of 3N HCl to each well, and the presence of proteins into which 5-(biotinamido) pentylamine had been incorporated was quantified by measuring the absorbance at 450 nm in a plate reader (Molecular Devices). Enzyme activity is expressed as specific activity.

### *In situ* labeling of TGase activity

Cultured cells were seeded on coverslips, and maintained in six well plates. Then the cells were labeled with 1 mM 5-(biotinamido)-pentylamine (BPNH_2_; Molecular Probes, Pierce) for 12 hours, and subsequently fixed according to Balajthy et al. [25]. Briefly, the cultured cells were fixed with 4% paraformaldehyde in HEPES (4°C; 10 minutes); 8% paraformaldehyde in HEPES (4°C; 50 minutes); 4% paraformaldehyde in HEPES (4°C; 20 minutes), 2% paraformaldehyde in PBS (15 minutes), and then in 100% methanol (-20°C; 20 minutes). After fixation, the cells were incubated in 25 mM glycine in PBS (20 minutes); permeabilized with 0.1% Triton X-100 in PBS (20 minutes); washed five times for 20 minutes in TTBS; blocked with TTBS, 5% BSA, pH 7.4 (20 minutes); incubated (1 hour) with Streptavidin-FITC Conjugate (GE, 1:200); washed 5 times for 30 minutes in TTBS; and then rinsed three times in PBS before coverslips were mounted in Mowiol (Vector Labs, Burlingame, CA) dissolved in glycerol.

### BrdU incorporation assay and transglutaminase activity

Cultured cells were seeded on coverslips, and maintained in six well plates. Then the cells were labeled with 1 mM 5-(biotinamido)-pentylamine (BPNH2; Molecular Probes, Pierce) and 10 μM BrdU (Sigma) for two or sixteen hours. The labeled cells were fixed as described above, and was subsequently incubated in 2N HCl containing 0.2 mg/ml pepsin (Sigma) for 30 min at 30°C, and washed with 0.1 M Na_2_B_4_O_7 _(pH 8.5) to neutralize the acid, and after washing with PBST (PBS with 0.5 % Tween) 3 × 5 min at 20°C, blocked with PBST, 5% BSA, pH 7.4 (20 min), incubated one hour with FITC-conjugated anti-BrdU (Becton Dickinson 1:50) and Cy5-Streptavidin (GE healthy, 1:1000). Then the coverslips were washed five times for 30 minutes in PBST; and then rinsed 3 times in PBS before coverslips were mounted in Mowiol (Vector Labs, Burlingame, CA) dissolved in glycerol. In order to detect cell surface TGase activity some cells were analyzed for TGase activity by omitting the permeabilization step with Triton X-100.

## Results

### TGase is abundant in Hpt cells

**Figure 1:**
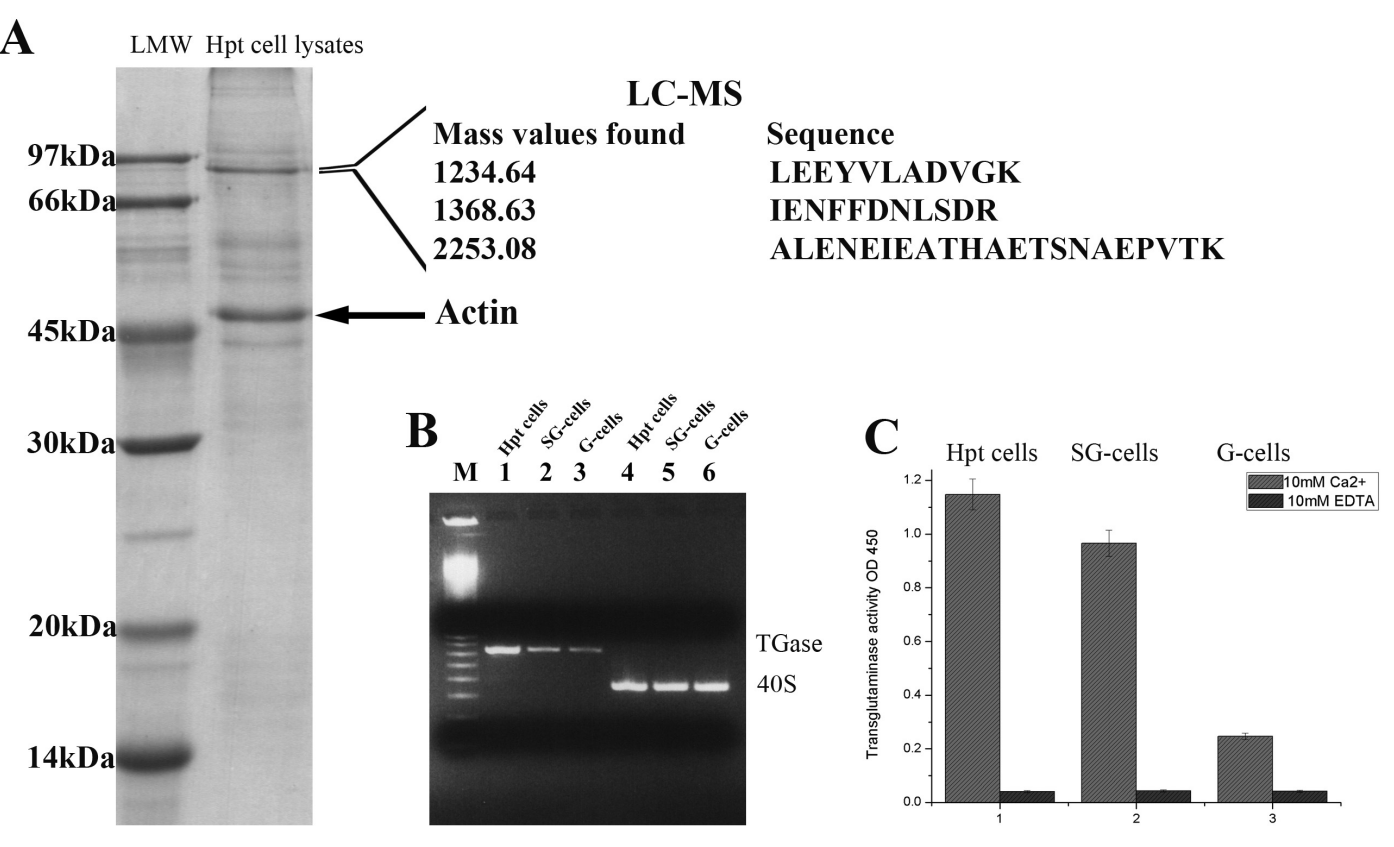
**TGase is an abundant protein in Hpt cells**. A. Hpt cell lysate separated by 12.5% SDS-PAGE. The main protein band around 90 kDa was confirmed to be the crayfish TGase by LC-MS. The strong band at 50 kDa (arrow) was confirmed to be actin. B. TGase transcript levels in Hpt cells and in separated circulating SG and G hemocytes analyzed by RT-PCR. Control transcript is 40S ribosomal protein. Lane 1, Hpt cell TGase; lane 2, SG-cell TGase; lane 3, G-cell TGase; lane 4, Hpt cells 40S; lane 5, SG-cell 40S; lane 6, G-cell 40S; M is 100 bp DNA marker. C. TGase activity in Hpt cell lysate and in lysate from separated SG or G hemocytes. The amount of incorporated 5' (biotinamido) pentylamine was quantified by measuring the absorbance at 450 nm in a plate reader. Mean ± SD of 3 separate experiments.

TGase has earlier been shown to be an important enzyme in the clotting reaction of crayfish and other crustaceans [4], and here we report that TGase (GeneBank accession no. AF336805) is one of the most abundant proteins in the hematopoietic tissue cells of crayfish. When Hpt cell lysates were separated by SDS-PAGE, two main protein bands were observed, one with a mass of about 90 kDa, which was confirmed by LC-MS to be the crayfish TGase (Fig. 1A) and the other protein is was confirmed by LC-MS to be crayfish actin (GeneBank accession no. DQ874398). The question why TGase is very abundant in the Hpt cells led us to study its function in the Hpt. First, we compared the TGase gene expression in Hpt and in the main two types of hemocytes in the circulation namely the semi-granular hemocytes and granular hemocytes. As shown in Fig. 1B, a gradual decrease in expression of TGase mRNA from Hpt cells, semigranular (SG) hemocytes and granular (G) hemocytes was detected by RT-PCR. The transcript levels were in accordance with the TGase enzyme activity assayed with 5'(biotinamido)-pentylamine as substrate showing high enzyme activity in Hpt cells and lower in SG cells and in the G cells the activity was very low (Fig. 1C).

### 5' regulatory region of Crayfish TGase contains one intron and several GATA binding motifs

**Figure 2:**
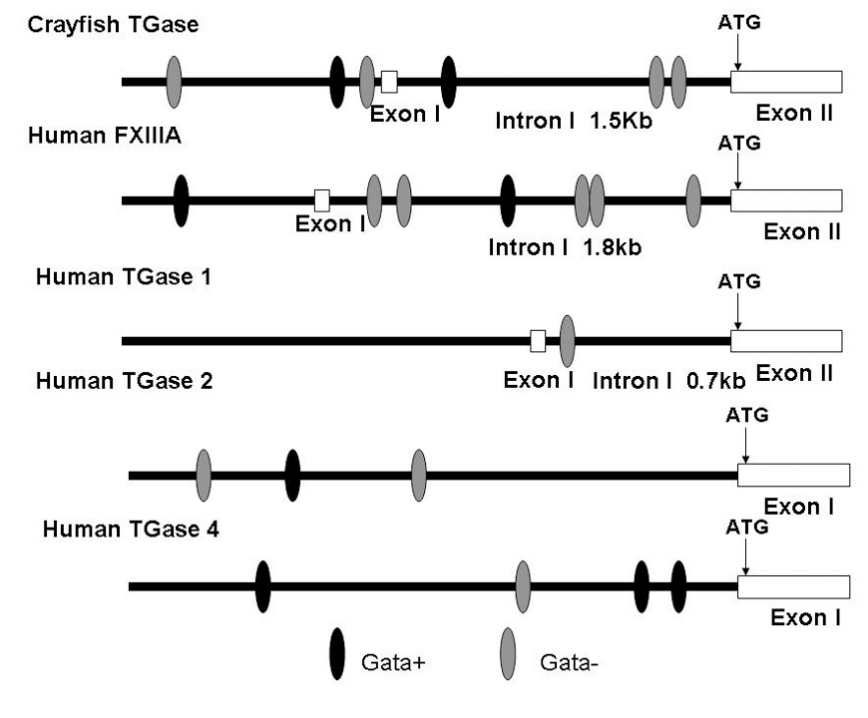
**The promoter region of TGase contains GATA motifs**. Schematic diagram of the 5'-flanking regulatory sequences and distribution of GATA motif in crayfish TGase and some human TGase genes.

As shown above transcription of the TGase gene was down regulated in differentiated hemocytes as compared to the Hpt cells, and the activity assay likewise indicates a high activity in the proliferating cells inside the tissue. With the aim of finding any regulatory regions of the TGase gene, a genome-walking procedure was performed in the 5' region of the crayfish TGase gene. A 2693 bp fragment was isolated and sequenced (GenBank accession no. EU195879) and the structure of this region was found to have similarities with mammalian plasma factor XIIIa and TGase I [26,27], showing one intron before and a translation start codon in the second exon (Fig. 2). Furthermore, in silico analysis of the sequence showed that the 5' regulatory region of crayfish TGase contains a series of GATA binding motifs, which also is present in the promoter region of factor XIIIa [26]. Since GATA transcription factors are known to be essential for the hematopoietic process in mammals as well as in invertebrates [19], a role for GATA factors in the regulation of expression of the TGase gene is possibly also of importance in crayfish hematopoiesis.

### TGase activity disappears when Hpt cells start to migrate

**Figure 3:**
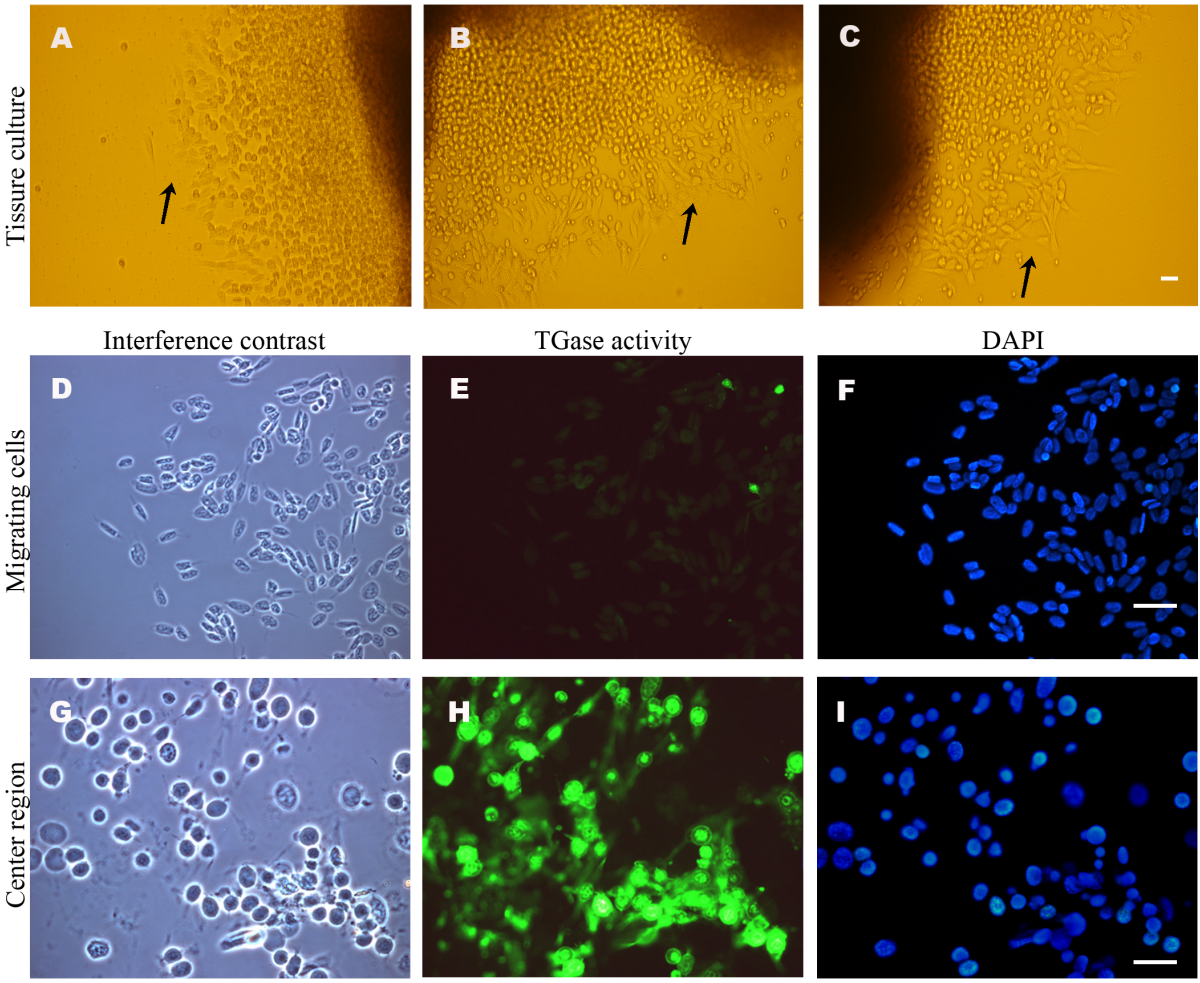
**TGase activity is low in cells migrating out of the Hpt**. Hematopoietic tissues were cultured on coverslips for one week, and labelled with 1 mM BPNH_2 _overnight. A-C. Migrating (spread) cells (arrows) can be seen at distance from the centre of the tissue; D-F. TGase activity in migrating cells; G-I. TGase activity in cells close to the centre of the tissue. Interference contrast (D, G); TGase activity (E, H); Nuclear staining was performed with DAPI (F, I). Bar is 40 μm.

**Figure 4:**
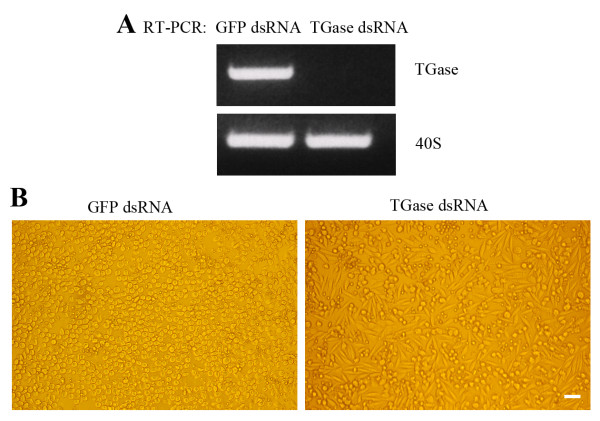
**RNA interference of TGase in cultured Hpt cells**. A. TGase expression analyzed by RT-PCR, five days after transfection with TGase dsRNA or GFP dsRNA (as control). 40 S ribosomal protein was used as a control transcript. B. Morphology change of Hpt cells transfected with dsRNA for TGase. Left, control cells transfected with GFP dsRNA; right, cells transfected with TGase dsRNA. Bar is 40 μm.

The hematopoietic tissue in crayfish contains cells of different developmental stages on their way to mature hemocytes [27], and in order to find out whether there is a difference in TGase activity between these stages, *in situ* labelling of TGase activity using pentylamine as substrate was performed. After one week in culture, cells from inside of the tissue had migrated out onto the coverslips surface. At a certain distance from the centre of the tissue the migrating cells started to spread (Fig. 3A–C). As shown in Fig. 3G–I, high TGase activity was present in cells close to the centre of the tissue, while in spreading cells at the migrating front TGase activity was hardly detected (Fig. 3D–F). In order to elucidate whether this lack of TGase activity was connected to the change in morphology, RNAi experiments were performed in *in vitro *cultured Hpt cells. By incubating Hpt cells with dsRNA for TGase, the transcript level was highly decreased after three days and was silenced completely after five days (Fig. 4A). Concomitant with the decrease in TGase transcription a change in the cell morphology was observed and about one third of Hpt cells started to spread intensely (Fig. 4B), indicating that the presence of TGase transcript is involved in preventing spreading of the Hpt cells.

### TGase activity does not affect proliferation of Hpt cells

**Figure 5:**
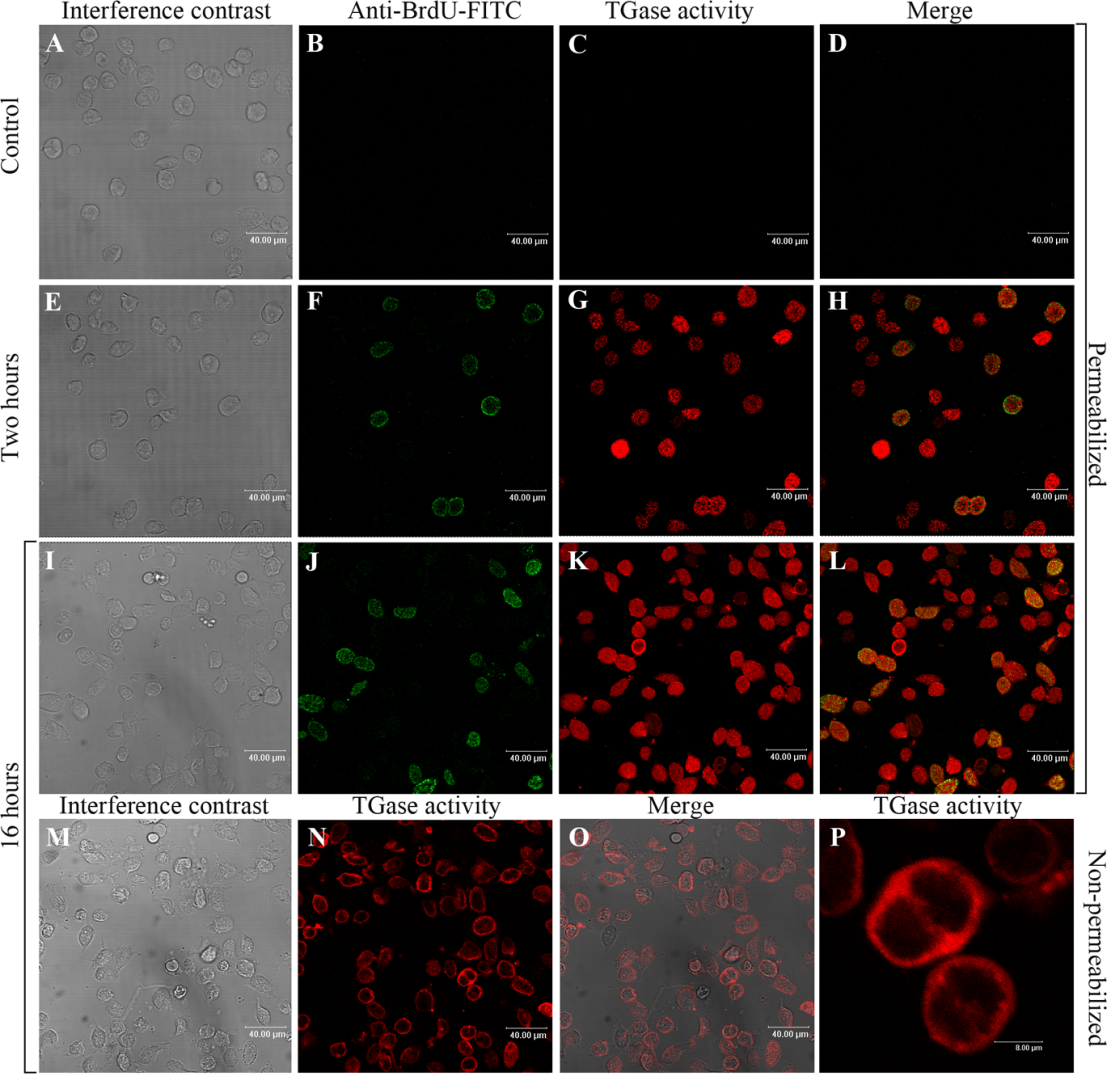
**TGase activity of HSCs does not prevent its proliferation**. Hpt cells were cultured on coverslips, and maintained in six well plates. The cells were labeled with 1 mM 5-(biotinamindo)-pentylamine (BPNH_2_; Molecular Probes, Pierce) as a substrate for TGase and 10 μM BrdU (Sigma) for 2 or 16 hours. The *in situ *TGase activity was stained by streptavidin-Cy5, BrdU incorporation assay was measured by staining with anti-BrdU-FITC. A-D. Control cells incubated without BrdU and BPNH_2._E-H. Cells incubated with BrdU and BPNH_2 _for 2 hours. I-L. Cells incubated with BrdU and BPNH_2 _for 16 hours. M-P. Cell surface TGase activity detected in non-permeabilized cells after incubation with BPNH_2 _for 16 hours.

Mammalian TGase 1 was found to contribute to terminal differentiation of keratinocytes [2], and the induction of enzyme activity of this TGase usually is initiated concomitant with cease of cell proliferation. In order to investigate whether high TGase activity in Hpt cells is accompanied by stop in proliferation, BrdU incorporation assays were performed for a short time (2 hours, Fig. 5E–H) and for a longer period (16 hours, Fig. 5I–P). However, in contrast to mammalian TGase 1 in keratinocytes, crayfish TGase activity was high in proliferating Hpt cells (Fig. 5E–L). Furthermore, to elucidate whether surface TGase was present in proliferating Hpt cells we repeated the experiment described above using non-permeabilized cells. As shown in Fig. 5M–P, the majority of cells show surface localized TGase activity, and especially cells undergoing mitosis showed extremely high TGase activity on the cell surface (Fig. 5P). These results taken together show that crayfish TGase activity is not initiated by stop in cell proliferation, as is the case in mammalian keratinocytes.

### Astakine induces spreading and loss of TGase activity

**Figure 6:**
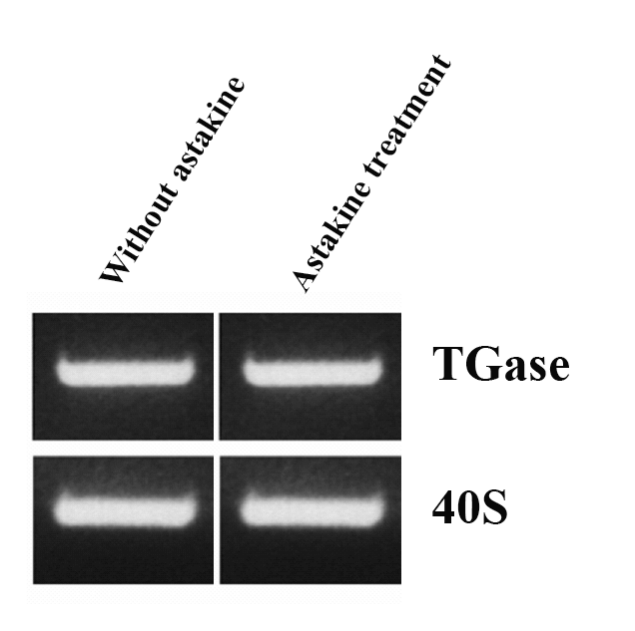
**Astakine does not affect TGase mRNA expression**. TGase expression analyzed by RT-PCR, four days after culture in the presence or absence of astakine. 40 S ribosomal protein was used as a control transcript.

**Figure 7:**
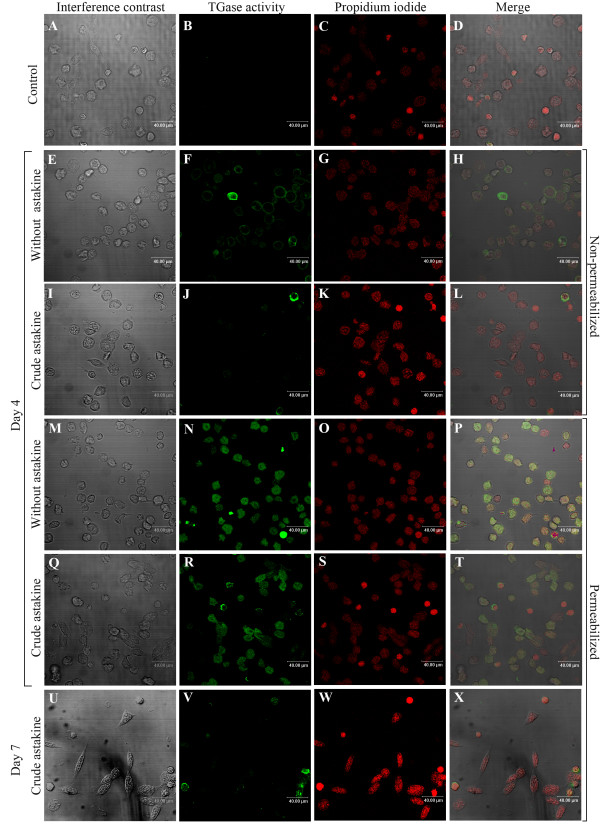
**Astakine treatment results in decreased TGase activity in the Hpt cells**. Hpt cells were seeded on coverslips, and maintained in culture dishes, and the medium supplied was added with or without astakine (a crayfish prokineticin domain-containing protein) [21]. At day 4 (E-T) and day 7 (U-X), the cells were incubated with 1 mM BPNH_2 _in medium two hours at room temperature, and visualized using the streptavidin-FITC conjugate. Nuclear staining was performed with propidium iodide. A-D. Non-permeabilized Hpt cells cultured for 4 days without BPNH_2 _were used as a staining control. E-H. Non-permeabilized Hpt cells cultured for 4 days without astakine showing surface TGase activity. I-L. Non-permeabilized Hpt cells cultured for 4 days with astakine showing no surface TGase activity. M-P. Permeabilized Hpt cells cultured for 4 days without astakine. Q-T. Permeabilized Hpt cells cultured for 4 days with astakine. U-X. Permeabilized Hpt cells cultured for 7 days with astakine showing no TGase activity. Bar is 40 μm.

In the Hpt cell culture, when the medium is supplemented with astakine (a crayfish prokineticin domain-containing protein) partly purified from crayfish plasma, from the third day, many cells start to spread and adopt morphology changes similar to that observed after TGase RNAi in the Hpt cell cultures. Accordingly, we investigated whether astakine treatment resulted in a similar loss of TGase activity in spread cells. First we analyzed transcript levels of TGase mRNA in astakine treated cells and could show that astakine had no effect on TGase expression (Fig 6) Interestingly, after three days in culture in the presence of astakine, TGase activity was not detected at the cell surface of non-permeabilized cells (Fig 7A–D). In contrast cells cultured without astakine showed TGase activity located on the outside (Fig 7E–H). When treated with astakine, spreading cells showed a gradual decrease of intracellular TGase activity (Fig. 7Q–T), and after about one week these avidly spread cells completely lost their TGase activity both inside and outside the cells (Fig. 7U–X). In order to reveal whether astakine inhibits TGase activity on the surface of Hpt cells, the surface TGase activity was assayed 5 hours after astakine addition. However, astakine induced decrease in extracellular TGase activity was not detected until 2 days after the addition indicating an indirect effect of astakine on TGase release from the cells.

## Discussion

Crustacean TGases have mainly been implicated in hemolymph clotting and wound healing [4-9] and in the freshwater crayfish, *P. leniusculus *TGase has earlier been shown to be responsible for the Ca^2+^-dependent cross linking of the clotting protein, a high-density lipoprotein present in plasma [4]. All crustacean TGases known so far show highest similarity with mammalian plasma factor XIIIa. Furthermore, shrimp TGase was shown to be present in plasma as weakly associated dimers in a similar way as for Factor XIIIa [28]. In the present study we also confirm this similarity by showing that the 5'regulatory region of the crayfish TGase gene is similar to the corresponding region of the Factor XIIIa gene. The presence and location of intron 1 as well as the presence of several GATA binding motifs are common characters of these two genes, suggesting a common ancestral origin of Factor XIIIa and crayfish TGase. The vertebrate TGase gene family is speculated to have developed from a common ancestral gene [29]. In shrimp two different TGases are identified [6], but it seems as if crayfish only express one TGase gene similar to that in several other invertebrates such as in *Drosophila melanogaster *and *Caenorhabditis elegans*.

When we analyzed the expression of TGase in Hpt cells, SG-cells and G-cells, a gradual decrease in transcript level as well as enzyme activity was revealed, showing a very high level of TGase mRNA and activity in Hpt cells and low mRNA levels and activity in the G-cells. Similarly high TGase expression assayed by *in situ *hybridization was detected in the tiger shrimp *P. monodon *hematopoietic cells as compared to circulating hemocytes by Huang and co-workers [17]. Collagenase type I and IV are used in order to dissociate Hpt cells from the lobules for use in *in vitro *culture experiments [21] and collagen is well known as a substrate for TGase [16]. This means that collagen most likely is one important ECM protein in the hematopoietic tissue, and can be used as a substrate for TGase. Keeping the cells in a close connection to each other by the ECM network may be needed for Hpt cells renewal, and consequently knockdown of TGase resulted in loss of the matrix network in the cultured cells and these cells could then start to migrate into the hemolymph.

When the TGase transcript was silenced in our experiment, the Hpt cells started to spread, mimicking the migration of cells out of the hematopoietic lobules, in agreement with what could be seen after *in vitro *incubation of the hematopoietic tissue. Simultaneously, concomitant with cell spreading the TGase enzyme activity disappeared. Interestingly, treatment of *in vitro* cultured Hpt cells with crude astakine (a crayfish prokineticin domain-containing protein) [21] likewise induced spreading and loss of surface localized TGase activity, indicating that there is a link between astakine treatment and TGase activity. A similar disappearance of cell surface TGase (Factor XIIIa) activity is known for mammalian monocytes during their differentiation into macrophages [30] indicating a related role for TGase in mammalian macrophage differentiation. This result shows a striking resemblance with recent reports on prokineticin 1 (PK1/EG-VEGF) effects on differentiation of mammalian monocytes, showing increased spreading and differentiation into macrophages [31], and will hopefully encourage more studies about the relation between prokineticins and TGase activities.

## Conclusion

In conclusion our results suggest that TGase is important for keeping the Hpt cells in an undifferentiated stage inside the hematopoietic tissue and if expression of TGase mRNA is blocked the cells start to migrate out of the tissue. This shows a new function for transglutaminase in preventing hematopoietic stem cells from being released into the hemolymph, whereas their proliferation is unaffected. Astakine is also important for the hematopoiesis since it induces new hemocyte synthesis in the Hpt and rapid release of new hemocytes, but now we also show a role for astakine in regulating TGase activity in the hematopoietic tissue and thereby affecting recruitment of new hemocytes to the circulating hemolymph.

## Authors' contributions

XL carried out most of the experiments, participated in experimental design and in interpreting the data and writing the manuscript.  IS participated in designing experiments, interpreting the data, drafting and writing the manuscript.  KS participated in designing experiments, interpreting the data, drafting and writing the manuscript.
